# Project Aedes transgenic population control in Juazeiro and Jacobina Bahia, Brazil

**DOI:** 10.1186/1753-6561-8-S4-O11

**Published:** 2014-10-01

**Authors:** Aldo Malavasi

**Affiliations:** 1 Moscamed Brasil, Juazeiro, Bahia, Brazil

## 

The genetic control of insects is a promising technology to be applied for the control of wild populations in urban and agricultural areas as an alternative to the widely used methods based on chemical and mechanical control. In Brazil, there is considerable concern about the occurrence of Dengue disease across the country, and a clear demand for improved vector control. A joint project among Medfly and Mosquito Facility Brazil, University of São Paulo and Oxitec was agreed upon in order to test in urban areas, social, technical and operational aspects of genetic control for *A. aegypti *population employing an RIDL transgenic strain. The PAT - Project Aedes Transgenic - was established in the city of Juazeiro, by a demand of Secretary of Health of State of Bahia. As the first large project on releasing transgenic insets in a human populated area, the PAT would follow all phases and steps recommended by the international community and fit into the strict Brazilian biosecurity laws.

Community engagement - From the outset the project partners have worked closely with the Brazilian regulatory system to obtain required permits for field activities. It has been a clear focus of PAT from the outset to adopt full transparency and a vigorous and proactive community engagement campaign.

Mass rearing **- **Critical to any SIT program is the ability to consistently rear large volumes of high quality insects. Egg production has an average of 12 million/week and 700,000 males. Field activities have concentrated on the suburb of Itaberaba. Males were marked with different colors and released from the same point in order to evaluate relative dispersal and longevity. The RIDL strain dispersed further with a mean distance travel of 75m (95% CI 30-109) compared to wild Itaberaba strain, 44m (95% CI 20-73), although this difference was not significant. Ovitrapping formed the main monitoring tool for *A. aegypti *- wild populations in both the treated and the untreated areas of Itaberaba. Larvae hatched from field-collected eggs from ovitraps were screened for fluorescence to determine paternity (fluorescent larvae, transgenic father; non-fluorescent larvae, wild-type father). Adult trapping was conducted periodically using aspiration surveys and BG-traps. This coincided with the release of marked cohorts of RIDL mosquitoes enabling longevity of released males to be assessed. After 12 months of twice week releases - 18 millions - the suppression reached 96%. In another district - Mandacaru - the releases started in march 2012 and with the density of 14,000 males/ha/week in 5 months, 100% of larva captured in ovitraps were fluorescent, indicating a high suppression. After the success in both districts, a larger target was aimed, a urban population of 45,000 people, city of Jacobina, where the releases started in June 2013 with a perspective to reach a large suppression in 24 months.

